# Government (Teaching) and Municipal (Non-Teaching) German Hospitals


**Published:** 1912-11-09

**Authors:** Henry Burdett


### GOVERNMENT (Teaching) and MUNICIPAL (Non-Teaching) GERMAN HOSPITALS.*

By SIR HENRY BURDETT, K.C.B.

### V. The Growing Cost of State Hospitals and its Consequences.

Last week we stated our intention to show that, as a matter of business, the cost of the great municipal hospitals in Germany is becoming so onerous that opinion in that country is looking with longing eyes in the direction of the voluntary system of hospital support with the hope and intention of relieving the municipalities of a portion at any rate of the burden these municipal hospitals entail upon the ratepayers. At the outset it may be well to make it clear that this view of the trend of opinion in Germany in regard to State hospitals does not emanate from the writer, but is the deliberate opinion of Herr Ober-Inspektor Einert, which was supported by a meeting of the Society of Chief Administration Officials of German Hospitals held in Dresden in 1911. We propose, therefore, to examine the grounds upon which the Chief Administration Officials of German Hospitals base the opinion just expressed.

### The Cost per Bed.

It has become every year more apparent that the burden which the German municipalities have taken upon themselves in building and maintaining the general hospitals increases by leaps and bounds. At the time this work was first undertaken by municipalities a hospital could be built at a cost of about £250 per bed. At the present time the cost in this connection of the new municipal hospitals is not less than £500 per bed. This is demonstrated by the figures published in connection with the expenditure upon buildings of some of the more recent hospitals, including that of Schwabing at Munich, where the actual cost is stated to have amounted to £535 per bed. Another example is that of the Johannstadt Hospital, Dresden, opened in 1901. The area of the site is 63,450 square metres and this hospital had in 1911 580 beds. The cost of construction was £475 per bed, which was increased to £510 per bed when the cost of fitting up the hospital and preparing it for opening—say £20,000—was added. In striking confirmation of the increase in the burden cast upon the municipalities which supply new general hospitals to the community in Germany at the present time, we may point to the case of another and older hospital at Dresden, the Friedrichstadt there, dating from 1849. The area of the site of this hospital is 101,000 square metres, the number of beds 1,272, and the cost of construction as entered in the Town Property List at the end of 1909 was £183 per bed against £475 per bed, which the new Johannstadt Hospital has cost for construction.

Take, again, what are known as the administration expenses in Germany. These include: The price of land for the site of municipal hospitals. The cost of the necessary materials for buildings and administration. The cost of drainage and modern hygiene requirements, and of the great developments in regard to equipment in all departments. This last necessarily includes everything pertaining to the practice of medicine and surgery under modern conditions, and the expensive apparatus and new departments and procedure which modern treatment in the highest type of hospitals entails. Add to the foregoing the outlay on,—The changed and ever varying methods of treatment which are frequently most costly. The organisation and maintenance of an adequate system of training and instruction for men and women attendants on the sick, and for the members of the scientific and general staffs of the hospitals. The improvement in the status and the enormous growth in the amount of salaries and wages paid to the staff of all grades in the highest type of hospitals in Germany to-day. The improvements in the equipment and accommodation of all departments of a modern hospital, and the multitude of other smaller, but, having regard to cost, considerable improvements and facilities which show a tendency to increase year by year. Individually these items represent material growth in cost, and collectively they total a sum which adds approximately something like one hundred per cent. to the cost per bed at the present time compared with that of twenty years ago.

### Why the Modern Hospital is Costly.

Let us examine these items a little in detail. A municipal hospital in Germany to-day means an extensive site capable of providing large and attractive grounds with well-kept and in many cases beautiful gardens, and the placing of each pavilion which contains patients in conditions which will secure for its inmates the maximum of light and air. In this connection Dr. Goldwater, of the United States, who, we conclude from his recent paper on "Hospital Planning," cannot be familiar with the latest and best specimens of municipal hospitals in Germany, states, in our judgment without adequate justification, that in that country there is manifested to-day a decided disposition to get away from the hospital of many widely separated but uniform pavilions. The view here expressed by Dr. Goldwater has, we venture to say, little or no foundation in fact. It is true, of course, that in the most recent and best of the municipal hospitals the scattered pavilions can be readily approached in bad weather by an underground subway of ample proportions and excellent ventilation, where all the various pipes and apparatus connected with heating, ventilation, water, lighting, and other

* Previous articles of this series appeared in our issues of October 12, 19, 26, and November 2.

essential hygienic and necessary systems are placed; but this fact only partially disposes of Dr. Goldwater's point. His general statement may possibly be due to the plan upon which the new and relatively vast medical pavilion now in course of completion in connection with the Royal Charité Hospital, Berlin,—an Imperial or State hospital as opposed to a municipal hospital,—has been conceived and is being built at the present time. A study of this plan and an inspection of the actual portions of it which are already in use convince us that it is a method of construction which should everywhere be avoided so far as possible.

### The German Pavilion System.

The reason for this particular method of one great building planned on what is known as the straight type is not, as Dr. Goldwater intimates, due to the hygienic necessities created by the unhygienic conditions and the undeveloped sanitary science of forty or fifty years ago, but to the circumstance that the Royal Charité is an Imperial hospital; and that it constitutes the great medical school and clinic where the medical staff of the German Army and Navy, present and in the making, are trained for their work and taught their business. In truth, the great and most recently planned and perfected municipal hospitals in Germany are admirable from the circumstance that, they are built upon a system of widely separated but largely uniform pavilions situated in spacious grounds, which are made as attractive and health-giving as it is possible to make them. Here, at any rate, is expressed the humane instinct to make the sick-house of to-day not only hygienically as perfect as possible for those acutely ill, but a place where every patient emerging from this condition is surrounded by everything which is calculated to inspire hope, and to stimulate and promote rapid recovery.

We therefore assert that it will be a grievous set-back in hospital planning if the United States of America, in the erection of their future hospitals, are to adopt a system of construction which gives a great mass of buildings tier above tier in one block, or the modified adoption of a similar idea, the linking together of pavilion after pavilion of hospital wards without any disconnection between them for the free circulation of air, as in the new buildings at the Royal Clinic in Berlin. Were they ever to fall into this grave error of construction the people of the United States would exhibit a total disregard, from the humane aspect, of the just claims of every patient whose circumstances necessitate his admission to a public hospital. Further than this it is the undoubted fact that the heap-of-buildings plan just referred to for town hospitals entails the erection outside the cities, where hospitals so constructed have been erected, of after-care establishments or convalescent institutions. Dr. Goldwater's Town Hospital to be efficient must have these costly additions to which patients with open wounds can be removed from the town hospital after the sixth or seventh day from the date of an operation, for that is about the time when surgical cases in buildings of this type in great cities often show the need for the greater vitality which good air supplies, to enable them to recover as quickly as possible by the rapid healing of their wounds and the building up of their constitutions.

### The Hospitals and Slow Recoveries.

Otherwise, or unless these town hospitals are associated with ample accommodation in after-care establishments, many patients must be discharged from the hospitals, as is often the case now, in a condition of health which forbids their return to their ordinary avocations, and may even compel them in considerable numbers speedily to return to the hospitals in shattered health for further treatment. In fact one of the great merits of the German idea in planning municipal hospitals is to give each member of the working classes who enters the wards the maximum chances of speedy recovery. The aim is to retain a patient in the favourable hygienic conditions we have already described under hospital care until he is sufficiently well, in the majority of cases, to resume his ordinary occupation in restored health. We make bold to say and to think that no hospital anywhere is entitled to rank as a modern, up-to-date hospital unless its organisation can guarantee to the worker the speediest possible recovery. Everything about it should tend to secure his restoration to his family in a state of health which justifies his resuming his occupation under the most favourable conditions to enable him to reap at once the benefit of the fullest earning-power his capacity and physical strength permit under ordinary conditions. Anyone who reads what we have written can readily see how this standard of hospital excellence can be attained.

There can be no economy justly claimed by a hospital which shows a reduced cost per bed in its construction or working when it clearly fails to reach the high standard of excellence here laid down. The improved methods and hygienic conditions now in force in the hospitals of London represent a monetary value to its working-class inhabitants which is equal to a gain in the total wages earned by them in a given year of not less than the sum represented by the proceeds of one million working days at least.

### Where the Money Goes.

When we consider the methods of treatment, developed as they are in Germany to the highest scientific point, we come to a field where cost has a tendency to increase and multiply. In the matter of equipment, in the supply of apparatus, in the fitting up and multiplication of laboratories, in the excellence of the furnishing and everything which tends to make residence as an in-patient materially comfortable, no money is spared. The same applies to the planning and equipment of the pathological department, the baths, the gymnasium, and the quarters where the nursing and domestic staffs are housed. Especially is this so in the pavilions in which the large staff of medical men working in the hospital reside. In order to give an idea of this latter accommodation we have selected for illustration the dining room of the medical staff at the Rudolph Virchow Hospital, Berlin. The quarters of the medical staff in the best of the German hospitals are, as they ought to be, excellent in all respects; such excellence means the expenditure of money, but it also means a high average of health for the staff, and the best brains and knowledgeable energy in the work. Such qualities must be invaluable to the patients and the reputation of the institution.

Again, during the last twenty, and especially the last fifteen years, the standard of the workers and the rate of their remuneration has increased greatly. Thus the wages of a male nurse in 1884 in the Friedrichstadt Hospital was 675 marks, whilst in 1909 it had increased to 1,000 marks; and the highest salary of a male nurse there had grown from 870 marks in 1884 to 1,600 marks in 1909. The lowest salary paid to a female nurse there in 1884 was 591 marks and the highest 699 marks, whilst in 1909 these figures have increased to 800 marks and 1,100 marks respectively. In recent years the upward tendency in salaries is very apparent. Amongst the male staff of the porter class in the higher grades the wages ran in 1909 from 1,800 to 2,100 marks per year, whilst the great increase in the cost of nursing was further due to the fact that the number of nurses employed has nearly doubled in the twenty-five years, there being in 1884 8.4 patients per nurse, as compared with 4.8 patients per nurse in 1909, and in the better and newer type of municipal hospital there is now provided one nurse for every 4.2 patients. The remuneration of the medical staff has risen in the same period to the extent of 159.6 per cent., this increase being chiefly due to the development of the medical service in the hospitals. Thus, at the Friedrichstadt Hospital in 1884 there were nine principal and assistant practitioners with a daily average of 472 patients; whilst in 1909, with a daily average of 804 patients, the medical staff consisted of thirty-one principal and assistant practitioners; similarly in the same ratio this upward tendency is manifest throughout the establishment in regard to all ranks of people employed in the service.

We have not space to go into the details of this growth of expenditure through all the departments, though we should be glad to place the figures at the service of anyone who may be interested. Let it suffice to state that the working expenses of the hospitals to which these figures relate had increased in the twenty-five years, *i.e.*, in 1884 to 1909, by 94.1 per cent. It may be asked, Is this upward tendency in cost likely to continue, or may it not tend to average itself or even to decrease? Herr Einert's opinion is that it will not and cannot be arrested. His opinion is based upon the fact that the progressive development of social conditions in Germany will render it impossible that either the personnel or the material expenses of hospitals there can be uninfluenced thereby. In the matter of nursing alone, judged by the standard attained in England, trained nursing is yet to seek, so far as the majority of German hospitals are concerned. But State and municipal authorities are showing an ever-increasing interest in sick nursing, which must result in larger expenditure and a gradual improvement in the training and organisation of nurses throughout the country. Herr Einert we are glad to see looks forward to the time when there will be in German hospitals a standard of efficiency which will regulate the appointment and service conditions, both for practitioners and nurses in all the larger hospitals in Germany.

### A Great Hospital Fund from Voluntary Contributions.

Few German hospitals possess large invested funds. They are dependent for their revenue chiefly upon the fees paid by patients for treatment. As expenditure has grown, so the necessity for raising the fees charged to patients in all grades has increased too. This increase in the rates charged to patients has grown to the extent of 70.5 per cent. during the last twenty-five years in the Dresden hospitals. This growth is the more remarkable seeing that the taxable income of the inhabitants of Dresden in the period in question has only increased to the extent of 31.3 per cent. Despite this increase in the patients' fees the contribution by the Dresden municipality to its general hospitals increased from £3,983 in 1884 to £21,406 in 1909. Nor is this all, for the increased charge on the municipality grows steadily year by year. The case of Dresden is not due to any local or special causes, and the payments made by the Dresden municipality were exceeded by those paid to the smaller hospitals in other German towns of importance. Indeed Herr Einert declares:

"A higher degree of development to which the modern hospitals attain may precisely constitute a danger for the public health, and the public well-being, if the normal fees paid by patients have to be increased over and over again in correspondence with the increase in the working expenses of the hospitals, and many people should thereby be deprived of the possibility of going into a hospital." With a view to lessen this danger Herr Einert suggests "that it might be possible to build up a permanent endowment fund by carrying to its credit regular contributions, profit-earning municipal undertakings, such as savings banks, pawnbrokers, etc., by complimentary participations in lotteries, carnivals, and other public undertakings, which would supply contributions to the fund, and by bequests made for purposes of general public utility, by oblations, and inheritances falling to the State, etc."

His view is the more the knowledge of the growing cost of hospital maintenance, and its dangers, penetrates the public mind, the more it will be regarded not so much as a question of actual burden on the State or municipal treasuries, but of the general welfare and true charity, so that steps will be taken to prevent the exclusion of the less wealthy paying patients from the hospitals. He is therefore of opinion that voluntary contributions constitute a source of new revenue which should be organised in connection with the public hospitals of Germany, and that steps should be taken to raise by this means a hospital fund of considerable amount, with the purpose of obtaining free beds, and of paying the fees of many persons just above the Poor-Law standard. He quotes the results of private munificence in regard to hospitals in England and America, and advocates that the millions of pounds bequeathed annually for the public benefit in Germany should by strenuous efforts be induced to flow into the hospital fund. In this connection he declares it to be desirable that the hospitals should be provided from suitable sources with interest bearing capital investments, the income from which is to be used to cover income deficiencies, and especially the maintenance of a scale of moderate fees within the means of the humbler class of patients to whom hospital treatment is a matter of the first importance.

We hope that the facts contained in this article may be brought to the notice of those people throughout Great Britain who have shown of late a trend in the direction of municipalising our hospital system. We have shown in this and former articles that the present excellence of our voluntary hospitals, where the first interest is always the interest of the patient, cannot be equally safeguarded under the system of municipal hospitals. We have shown inferentially and definitely that the voluntary system which has provided the British nation with the most efficient and best system of nursing known to the world, which affords the maximum of protection and comfort to the patient, and a high efficiency in matters of treatment, is, from an economical point of view, also better than that of any other system of hospitals extant. Further than this, the burden of municipal hospitals tends to increase so rapidly that in Germany there is a marked trend of opinion in the direction of a strong national effort to secure voluntary contributions so as to lessen the present burden of hospital charges upon the ratepayers and the patients. These are provable facts, and we commend them to the earnest consideration of people of all classes resident within the United Kingdom.

*(To be continued.)*

